# Magnitude and Predictors of Antenatal Depression among Pregnant Women Attending Antenatal Care in Sodo Town, Southern Ethiopia: Facility-Based Cross-Sectional Study

**DOI:** 10.1155/2020/6718342

**Published:** 2020-04-01

**Authors:** Bekalu Thomas Chuma, Getu Gamo Sagaro, Feleke Hailemichael Astawesegn

**Affiliations:** ^1^Sodo Christian General Hospital, Wolaita Sodo, Ethiopia; ^2^School of Public Health, College of Health Sciences and Medicine, Wolaita Sodo University, Wolaita Sodo, Ethiopia; ^3^School of Public Health, College of Medicine and Health Sciences, Hawassa University, Hawassa, Ethiopia

## Abstract

**Background:**

Depression affects approximately 10 to 20% of pregnant women globally, and one in ten and two in five women in developed and developing countries develop depression during pregnancy, respectively. However, evidence regarding its magnitude and predictors in Southern Ethiopia is limited. The present study is aimed at assessing the magnitude and predictors of antenatal depression among pregnant women attending antenatal care in Sodo town.

**Methods:**

A facility-based cross-sectional study was conducted among 403 antenatal care attendants in Sodo town from November 2 to January 30, 2017. Systematic random sampling was used to select the study population, and data were collected by using a pretested and structured questionnaire. Data were entered using Epi-data 4.2 and then exported and analyzed using SPSS version 20. Bivariate and multivariable logistic regression analyses were used to assess the association between the dependent variable and independent variables. Variables with *P* value less than 0.05 were considered as statistically significant.

**Results:**

A total of 400 pregnant women were interviewed. The magnitude of antenatal depression was 16.3% (95% CI (12.8%, 19.9%)). Husband's educational status, at the college and above (AOR: 0.09; 95% CI (0.03, 0.34), regular exercise (AOR: 0.16; 95% CI (0.07, 0.36)), planned pregnancy (AOR: 0.16; 95% CI (0.06, 0.44)), use of family planning (AOR: 0.31; 95% CI (0.14, 0.66)), previous history of anxiety (AOR: 2.96; 95% CI (1.30, 6.74)), previous history of obstetric complications (AOR: 19.03; 95% CI (5.89, 61.47)), and current obstetric complications (AOR: 30.38; 95% CI (3.14, 294.19)) were significant predictors of antenatal depression.

**Conclusion:**

Nearly one in six pregnant women had antenatal depression. The husband's educational status, regular exercise, planned pregnancy, use of family planning, previous history of anxiety, previous history of obstetric complications, and current history of obstetric complications were significant predictors of antenatal depression. Screening for depression during routine antenatal care could be essential and recommended to identify early and prevent further morbidities and mortalities due to antenatal depression.

## 1. Introduction

Depression is one of the most prevalent and severe mental disorders affecting women [1–3]. Globally, it affects approximately 10% to 20% of pregnant women [4]. Most people usually recognize and are comfortable with a change in mood, but people with depression, however, often cannot explain the reason for becoming depressed, though they describe it as emotionally painful and saddening. The predominant symptoms of depression are a general loss of interest and energy and an inability to experience pleasure [5]. Antenatal depression, also known as prenatal depression, is a form of clinical depression that can affect a woman during pregnancy and can be a precursor to postpartum depression if not properly treated. Besides, one in ten women in developed countries and two in five women in developing countries develop depression during pregnancy [6–8].

Antenatal care traditionally focuses on physical health rather than on mental health. But psychological problems and mood disorders can occur during pregnancy and may have impacts on the lives of people, primarily when related to pregnancy [9]. Besides, it is highly related to poor obstetric and infant outcomes [10], has also shown to have impacts on offspring, has also been associated with a range of adverse offspring outcomes (higher risks of premature birth, low birth weight, intrauterine growth restriction, child emotional and behavioral problems, and cognitive difficulties) and later postnatal depression [11–14], and has been associated with an increased risk of adverse fetal health and negative effects on fetal neurodevelopment [15].

The prevalence of antenatal depression was high in African countries when compared to other countries around the world, and pregnant women living in developing countries and rural areas are more prone to depression than those living in developed and urban settings [16]. Different studies have documented that the prevalence of antenatal depression among pregnant women ranges from 11.8% to 47% [17–19]. Several studies had reported that antenatal depression was significantly associated with age [20], unplanned pregnancy [21], smoking during pregnancy, history of abortion and stillbirth, presence of a complication in the current pregnancy, previous history of depression, low educational attainment, and irregular menstrual history [17, 22–24]. Besides, domestic violence, financial stress, history of miscarriage, marital conflict, and drinking alcohol during pregnancy were significant predictors of antenatal depression [4, 25, 26].

Studies were conducted in different parts of Ethiopia and reported various findings of antenatal depression [8, 17, 27–31]. However, there is still a low integration of mental health services into primary care and low recognition rates by primary care health workers due to the shortage of clinicians with specialized training in assessing and managing women with mental disorders. Besides, antenatal depression among pregnant women was not considered a significant problem by policymakers. Therefore, the present study is aimed at assessing the magnitude and predictors of antenatal depression among pregnant women attending antenatal care in Sodo town, Southern Ethiopia. The results of this study provide possible interventions that can be used by the government and various stakeholders to prevent antenatal depression in pregnant women.

## 2. Materials and Methods

### 2.1. Study Setting and Period

The present study was conducted in the Sodo town of Wolaita Zone from November 2 to January 30, 2017. Sodo town is located 385 kilometers southwest from Addis Ababa, the national capital city, and 170 kilometers West of regional town, Hawassa, at 6°49′N latitude and 39°47′E longitude and at an altitude of about 1,900 m. Sodo town is the capital city of Wolaita zone, with an area of 830 square kilometers. The population of the city is projected to be about 137,522 for July 2014. The town is structured into three subcities and 11 kebeles (lowest administrative units).

### 2.2. Study Design and Participants

The facility-based cross-sectional study design was used, and pregnant mothers attending antenatal care from selected health facilities during the study periods were considered as the study participants. However, a pregnant mother who was seriously ill and unable to speak and/or hear were excluded from the study.

### 2.3. Sample Size Determination and Sampling Procedure

The sample size was calculated by using the single population proportion formula:

(1) $$\begin{array}{c} \frac{n=\left(Z\;1\left(\alpha /2\right)\right)2*p\left(1-p\right)}{d^{2}}, \end{array}$$

where *n* is the required sample size; *Z* is the critical value for normal distribution at 95% of confidence interval (CI), which is 1.96; *d* is the margin of error (5%); and *p* is the proportion of pregnant women having antenatal depression, and 50% was considered, and an estimated nonresponse rate was 10%. Accordingly, the calculated sample size was 403. The total sample size was proportionately allocated for each health facility depending on the average number of pregnant mothers who were visited antenatal care in a month before the start of this study. The study participants were identified by using a systematic random sampling method. Besides, the simple random sampling method was employed for the first participant to get the starting point and then depending on sampling interval (every other pregnant woman) participants coming to antenatal care to be enrolled from each selected facility until the required sample size was obtained.

### 2.4. Data Collection Process and Tool

Data were collected using a pretested and structured questionnaire. The questionnaire was first prepared by English and translated into Amharic by experts and then back to English to check for its consistency. Finally, the Amharic version of the questionnaire was used to collect the data. The antenatal depression was judged according to the well-known Edinburgh postnatal depression scale (EPDS) measurement for depression during pregnancy. EPDS screening tool was validated and approved for use in Ethiopia, and its internal reliability was tested (Cronbach alpha = 0.71) [16, 32]. The EPDS tool consists of 10 questions, and responses are scored 0, 1, 2, or 3 according to the increased severity of the case. Besides, items marked with an asterisk (∗) are reverse scored (i.e., 3, 2, 1, and 0). Finally, the scores from each point were summed up, and a score of 13 and above was used as a cutoff point to detect antenatal depression [9, 28].

Two female diploma midwives and one female nurse for data collection and two midwives with M.Sc. and B.Sc. for supervision were recruited. Data collectors and supervisors were trained for two days on the objectives of the study, ethical conduct of the research, on the questionnaire, EPDS measurement scale, and some other technical things, including role play. Besides, the questionnaire was pretested on 5% of the total sample size in the Boditi health center, which is located around 18 km away from the study area.

### 2.5. Study Variables

The dependent variable of this study was antenatal depression. Independent variables were sociodemographic factors (age, sex, educational level, economic status, marital status, ethnicity, and residence), psychosocial factors (support from husband, household decision-making status, community support, previous history of anxiety, and happiness by ANC service provided), obstetric factors (parity, trimester, current obstetric complications, previous obstetric complications, need of current pregnancy, pattern of antenatal care, and history of family planning use), lifestyle factors (cigarette smoking, alcohol use, chat use, drug use, exercise during pregnancy, compliance with counseling and drugs prescribed during pregnancy, and rest during pregnancy), and awareness factors (about depression during pregnancy, treatment, and effect of ANC follow-up in identifying and treating antenatal depression). The previous history of anxiety was defined as an event of anxiety disorders such as panic disorder or attack, agoraphobia, social phobia, generalized anxiety disorder, and obsessive-compulsive that occurred before the current antenatal visit.

### 2.6. Data Processing and Analysis

Data was cleaned, coded, and entered using Epi-data 4.2 software and then exported to SPSS version 20 statistical package for analysis. Descriptive statistics were used to explore the data about the relevant variable, and a binary logistic regression was used to assess the association between the outcome variable and independent variables. According to the EPDS score, pregnant women who scored 13 and above were coded as “1,” and those who scored less than 13 were coded as “0” for binary logistic regression analysis. Besides, the economic level of mothers was analyzed using the international wealth index [33]. Variables with *P* value < 0.2 during bivariate logistic regression analysis were considered for multivariate analysis to identify significant predictors of antenatal depression [17, 27]. Also, the Hosmer-Lemeshow goodness of fit test with the enter method was used to test for model fitness. Finally, variables with *P* value less than 0.05 were considered as significant predictors of antenatal depression.

## 3. Results

### 3.1. Sociodemographic Characteristics of Participants

A total of 400 pregnant women were interviewed and resulting in a response rate of 99.3%. The median age of respondents was 24, with a standard deviation of 4.23. Nearly 96% (382) of respondents had a formal education, and almost all 395 (99%) of them were married. Ninety-two percent (367) were from Wolaita, and 375 (94%) were residing in the urban area. Nearly 75% of the respondents were protestant religious followers. Approximately 73% of the participants were from the household with a middle level of economic status (Table 1).

### 3.2. Psychosocial and Lifestyle Characteristics

Almost all 394 (99%) of the respondents reported that their partners were supportive. A total of 217 (54.3%) reported that they had support from the community, and 271 (68%) of the respondents also told us that they had support from their family. Eighteen percent (73) had a previous history of anxiety, and about 4% (13) of the respondents reported that their partners denied paternity to the current pregnancy. Regarding awareness about the problem, a total of 67% (269) had never heard about antenatal depression. Among those who had heard, 122 (93%) thought seeking treatment was important for the management of antenatal depression. Besides, 312 (78%) of the respondents reported that they had very good compliance with the drug prescribed by health professionals, and 76% (306) respondents reported that they were doing regular exercise of which 291 (95%) reported that they did walking as one of their exercises. Nearly 87% (346) of the respondents said that they slept for six-ten hours a day on average.

### 3.3. Obstetric Characteristics

The median for having living children was one with a minimum of zero and a maximum of seven while 44% of the participants had no living children. Close to 53% (213) of the respondents were with gravidity of two to five while 65% (260) were in the third trimester during the study period. Mostly 91% (364) of participants reported that their current pregnancy was planned, nearly 3 in 5 (59%, 235) of women had used family planning, and around 3.5% (14) respondents reported that health professionals mistreated them during ANC follow-up (Table 2).

### 3.4. The Magnitude of Antenatal Depression

**Figure 1:**
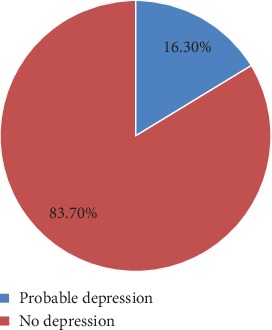
The magnitude of antenatal depression among pregnant women (*n* = 400) attending antenatal care in Sodo town health facilities, Southern Ethiopia, 2017.

In general, pregnant women who scored above the cutoff point in EPDS were 65 (16.3%), and participants who scored less than the cutoff point were 335 (83.7%). Accordingly, the magnitude of antenatal depression was 16.3%, with 95% CI (12.8, 19.9) (Figure 1).

### 3.5. Predictors of Antenatal Depression

Bivariate analysis showed that the husband's educational status, previous history of anxiety, regular exercise, previous history of obstetric complications, current obstetric complications, planned pregnancy, and use of family planning variables were having a *P* value less than 0.2. In the multivariate analysis, the husband's educational status specifically at the college and above, previous history of anxiety, previous history of obstetric complications, regular exercise, current obstetric complications, planned pregnancy, and use of family planning were found to be significant predictors of antenatal depression with *P* value < 0.05 (Table 3).

## 4. Discussion

The magnitude of antenatal depression among pregnant women in the present study was 16.3% (95% CI (12.8%, 19.9%)). This finding was lower than some studies conducted in another region of Ethiopia which reported 23% [27], 24.9% [28], 29.5% [29], 31.1% [8], 31.2% [30], and 31.5% [31] and higher than 11.8% [17] which was conducted in Debretabor town. However, the Debretabor study was community-based as well as the bale zone study was also not specific to pregnant mothers; it included postpartum mothers within one year after delivery. Besides, the magnitude of this study was also lower than that of studies conducted in other African countries [9, 15, 19]. These differences could be due to differences in geographical location, culture, socioeconomic status, the differences in cut off point for EPDS, screening methods of depression, and study setting. But the magnitude of this study was in line with a study conducted for low- and middle-level income countries which reported a weighted mean prevalence of 15.6% (95% CI (15.4%, 15.9%)) antenatally [34].

In this study, pregnant women married to husbands with the education level of college and above had 91% less chance to be depressed antenatally compared to husbands with no formal education. As there are a lot of psychological changes during pregnancy, it is obvious that the role of husbands during pregnancy is significant. The more the husbands are educated, they become more supportive of their wives and then the less their wives are depressed. This finding was in agreement with a study done in China [35]. Besides, this study also revealed that women who had the previous history of anxiety were 2.96 times more likely to develop antennal depression when compared to those women who had no previous history, and this finding was consistent with the studies conducted in KwaZulu-Natal, South Africa [9] and Debre Tabor, northwestern Ethiopia [17]. In addition, pregnant women who do regular exercise have 84% lower chance to be depressed during their pregnancy time than those who do not. A similar finding was reported from Sao Paulo, Brazil, which found that exercise during pregnancy was related to the reduced sign of depression [36].

The present study also found that pregnant women who had a history of obstetric complications were 19.03 times more likely to suffer from antenatal depression compared to those without previous obstetric complications. This finding was in agreement with the study conducted in Debre Tabor town, northwestern Ethiopia [17]. Besides, this study was reported that pregnant women with a history of current obstetric complications were 30.38 times more likely to develop antennal depression as compared to those who had no current obstetric complications.

Moreover, women who had planned their current pregnancy had 84% lower chance of developing antenatal depression than those who did not plan their pregnancy, and this finding was in line with a study conducted in Malaysia [21]. This might be explained that unplanned pregnancies are more likely to be unwanted, and unwanted pregnancies are the major causes of induced abortions which can cause many complications if not managed safely. Besides, an unplanned pregnancy can bring worries during pregnancy because women usually are not mentally ready to take care of the pregnancy in such circumstances [37]. Also, an unwanted pregnancy, especially during the premarital period has a negative attitude towards the current pregnancy [38, 39]. Consequently, this could lead to depression in pregnancy.

In this study, using family planning was found to be a significant predictor of antenatal depression, and those women who used family planning services were 69% less likely to have antennal depression when compared to those who did not use family planning services. This is possible because women who use family planning were more likely to plan their pregnancy, and that could help to decrease the chance of depression.

### 4.1. Limitation of the Study

Since it is a cross-sectional study design, the temporal relationship cannot be determined, and follow-up investigation may be needed to understand the nature of these factors in antenatal depression. Besides, this study was a facility-based study, and pregnant mothers who do not seek antenatal care services at selected health facilities would not be addressed. Moreover, antenatal depression was assessed using self-reported screening measures which are not a diagnostic tool and allow for possible recall bias.

## 5. Conclusions

Nearly one in six pregnant women attending antenatal care at health facilities in Sodo town had antenatal depression during the study period. Doing regular exercises, planned pregnancy, and the use of family planning was found to be significant protective predictors of antenatal depression. In contrast, the previous history of anxiety, previous history of obstetric complications, and current history of obstetric complications were significant risk factors of antenatal depression. Strengthening the existing program and increasing service utilization of family planning could help minimize depression in pregnancy. Besides, incorporating psychiatric services and routine preventive obstetric services as well as screening for depression during routine antenatal care would be very important and recommended to prevent further morbidities and mortalities due to antenatal depression.

## Figures and Tables

**Table 1:** Sociodemographic characteristics of pregnant women (*n* = 400) attending antenatal care in Sodo town health facilities, Southern Ethiopia, 2017.

Variables	Category	Frequency (*n*)	Percent (%)
Age	15-24	217	54.3
	25-34	171	42.7
	35-44	12	3

Educational status	No formal education	18	4.5
	Primary school	75	18.8
	Secondary school	168	42.0
	College and above	139	34.7

Marital status	Married	395	98.7
	Single	2	0.5
	Divorced	2	0.5
	Widowed	1	0.3

Ethnicity	Wolaita	367	91.7
	Amhara	9	2.3
	Oromo	2	0.5
	Gurage	3	0.8
	Others	19	4.7

Residence	Urban	375	93.7
	Rural	25	6.3

Religion	Protestant	294	73.5
	Orthodox	94	23.5
	Muslim	5	1.3
	Others	7	1.7

Occupation	Governmental worker	87	21.7
	NGO worker	52	13.0
	Merchant	25	6.3
	Student	10	2.5
	Daily laborer	62	15.5
	Housewife	164	41.0

Economic status	Low	71	17.8
	Middle	291	72.7
	High	38	9.5

**Table 2:** Obstetric characteristics of pregnant women (*n* = 400) attending antenatal care in Sodo town health facilities, Southern Ethiopia, 2017.

Variables	Category	Frequency (*n*)	Percent (%)
Previous obstetric complications	No	372	93.0
	Yes	28	7.0

Gravidity	Zero	169	42.3
	One-five	224	56.0
	Above five	7	1.7

Trimester	First	11	2.7
	Second	129	32.3
	Third	260	65.0

History of instrumental delivery	No	389	97.3
	Yes	11	2.7

History of stillbirth	No	384	96.0
	Yes	16	4.0

Current obstetric complications	No	389	97.3
	Yes	11	2.7

Planned pregnancy	No	36	9.0
	Yes	364	91.0

Family planning	No	386	96.5
	Yes	14	3.5

Living children	Below three	361	90.3
	Three-five	38	9.4
	Above five	1	0.3

**Table 3:** Predictors of antenatal depression among pregnant women (*n* = 400) attending antenatal care in Sodo town health facilities, Southern Ethiopia, 2017.

Variables	Antenatal depression		COR (95% CI)	AOR (95% CI)
	Yes	No		
Husband's educational status				
No formal education	16	10	1	1
Primary school	35	18	0.82 (0.31, 2.18)	0.84 (0.24, 2.99)
Secondary school	120	26	0.35 (0.14, 0.8)	**0.28 (0.09, 0.91**)^∗^
College and above	164	11	0.11 (0.04, 0.29)	**0.09 (0.03, 0.34)** ^∗^
Previous history of anxiety				
No	283	44	1	1
Yes	52	21	2.59 (1.43, 4.72)	**2.96 (1.30, 6.74)** ^∗^
Previous history of obstetric complication				
No	326	9	1	1
Yes	46	19	14.96 (6.39, 35.04)	**19.03 (5.89, 61.47)** ^∗^
Regular exercise				
No	68	26	1	1
Yes	267	39	0.38 (0.22, 0.67)	**0.16 (0.07, 0.36)** ^∗^
Current obstetric complication				
No	333	2	1	1
Yes	56	9	26.76 (5.63, 127.10)	**30.38 (3.14, 294.19)** ^∗^
Planned pregnancy				
No	16	20	1	1
Yes	319	45	0.11 (0.05, 0.23)	**0.16 (0.06, 0.44)** ^∗^
Use of family planning				
No	123	42	1	1
Yes	212	23	0.32 (0.18, 0.55)	**0.31 (0.14, 0.66)** ^∗^

^∗^Significant at *P* value < 0.05.

## Data Availability

The data that support the findings of this study are available on request from corresponding author.
